# Enduring hope and loss: qualitative evidence synthesis of LGBTQ+ experiences of perinatal loss

**DOI:** 10.3389/fpsyt.2025.1732197

**Published:** 2026-01-16

**Authors:** Zoë Josephine Darwin, Ash Bainbridge, Aikaterini Bekiropoulou, Mari Greenfield

**Affiliations:** 1 School of Human and Health Sciences, University of Huddersfield, Huddersfield, United Kingdom; 2 School of Nursing and Midwifery, University of Worcester, Worcester, United Kingdom; 3 Institute of Psychiatry, Psychology and Neuroscience, King’s, College London, London, United Kingdom; 4 Faculty of Wellbeing, Education and Language Studies, The Open University, Milton Keynes, United Kingdom

**Keywords:** LGBTQ+, perinatal loss, reproductive loss, qualitative evidence synthesis, perinatal care

## Abstract

**Introduction:**

Perinatal healthcare systems, services and research are shaped by cisheteronormative assumptions, i.e. that families involve one woman who carries a pregnancy and one man who is a non-carrying partner; furthermore, assuming that conception has usually resulted from sexual intercourse, with both parties providing gametes. These assumptions obscure and sometimes exacerbate LGBTQ+ people’s experiences and needs. This evidence synthesis aimed to identify and bring together the experiences of LGBTQ+ people who have faced pregnancy or baby loss; collectively perinatal loss.

**Methods:**

A qualitative evidence synthesis was conducted using systematic methods. Relevant databases were systematically searched using predefined search terms, and complimented by citation chaining. Eligibility was restricted to empirical qualitative studies published in English, unrestricted by participants’ relationship to the loss (i.e. physically pregnant or not - sometimes respectively described as gestational/birthing or non-gestational/non-birthing parent), type of perinatal loss (e.g. miscarriage, stillbirth), time since loss, setting, publication date, or type of qualitative methodology. Study selection followed a multi-stage screening process. Thematic synthesis was used to analyse and interpret patterns of meaning across included studies.

**Results:**

Seven studies met the eligibility criteria, reported across 10 papers. All seven were conducted in the Global North (including North America, Australia, and Europe). Thematic synthesis generated one overarching theme - enduring hope and loss – which captured the layers of loss experienced by LGBTQ+ people. This included the complexity of loss, and the loss commonly not being felt as an isolated incident, but rather part of a longer process. The three connected themes were: 1. Investment, which included the effort of navigating cisheteronormative systems, frequently after investing time, finances and emotions in assisted conception. 2. Support in relation to loss, highlighting the challenges of accessing support while being marginalised, excluded, or feeling invisible and, at times, unsafe as an LGBTQ+ family. 3. Meaning-making, in the immediate experience of loss, the aftermath of loss and the care received, and the time beyond.

**Conclusion:**

Cisheteronormative systems and interactions have potential to amplify loss and contribute to feelings of disenfranchisement amongst LGBTQ+ people. Further research is needed to evaluate support provided, inclusive of implications for subsequent reproductive choices.

## Introduction

Perinatal loss is an umbrella term used to include both pregnancy loss and baby loss. The language individuals use to describe their losses, which may also be experienced as bereavements, is not necessarily linked to the gestation at which the loss occurred. For example, some people may describe loss at an earlier gestation (including miscarriage and ectopic pregnancy) as *baby loss*, whereas others may describe this as *pregnancy loss*, reserving *baby loss* for later losses (including stillbirth and neonatal death). Similarly, language preferences cannot be assumed based on the clinical circumstances of the loss, including termination of pregnancy for any reason. Language preferences for how individuals identify themselves and identify other family members may also vary widely. This review therefore refers to *people* rather than assuming that all individuals who have experienced perinatal loss will identify as *parents*. This is not intended to diminish anyone’s identities, relationships or experiences. Where the term *parents* is used, it reflects the language used in the included studies.

Perinatal loss can carry considerable short and long-term emotional and psychological impacts. Physical pain during a perinatal loss is often accompanied by feelings of fear and helplessness (1). Initial post-loss feelings of guilt, shame and anxiety are common (1), as are grief responses, both for the person who has been pregnant and – where applicable – their partner. Some individuals will additionally be affected in the longer term by mental health difficulties that impact their day-to-day lives, relationships and functioning, including post-traumatic stress, anxiety and depression (2, 3). Assisted conception efforts that are ‘unsuccessful’ have been framed by some researchers as ‘reproductive loss’ (4) and are commonly accompanied by depression and anxiety in the short-term (5); these may also be experienced as perinatal loss.

Experiences in pregnancies subsequent to loss can also be impacted, for example through greater vulnerability to mental health difficulties (6). Vulnerability to subsequent parent-infant relationship difficulties may also be greater (7–9), and it is also important to normalise such difficulties (10). Whilst most research has been conducted with women who had carried the pregnancy, research with men (sometimes framed as partners, fathers or both) is now growing. Reviews of men’s experiences note challenges relating to lack of recognition and validation of their experiences, and reduced opportunities for access to support via healthcare services (11, 12). These indicate the importance of understanding support needs and making support available that responds to the needs across genders and across roles within families.

Services are increasingly providing care to sexual and gender minorities who are pursuing pregnancy and parenthood. This likely reflects both increased visibility and increased access to assisted conception (although this may not necessarily be required), accompanying changes to social and legal context in some countries. Research in this area is relatively recent and has previously focused on sexual minorities (LGB) whilst more recently gender minorities have been recognised. In this review, we use the term LGBTQ+ in the absence of a globally agreed more expansive acronym, noting differences for example between North American and European research.

Despite the increase in visibility of LGBTQ+ people pursuing pregnancy and parenthood, perinatal health care systems and services continue to be shaped by cisheteronormative assumptions, i.e. that families involve one woman who carries a pregnancy and one man who is a non-carrying partner; furthermore, assuming that conception has usually resulted from sexual intercourse, with both parties providing gametes (13–15). Additionally these systems and services are shaped by endonormativity, i.e. that endosex bodies (people whose sex characteristics fit typical binary notions of male and female) are the norm, erasing and pathologising intersex bodies. Where perinatal research does consider intersex bodies, this typically relates to babies with variations in sex characteristics. This range of assumptions may also bring significance for the extent to which relationships and needs are recognised within services. To-date, no reviews are available on LGBTQ+ people’s experiences of perinatal loss, precluding greater understanding, identification of need and provision of support. The purpose of this evidence synthesis was therefore to address this gap by answering:

What are the experiences of LGBTQ+ people who have faced perinatal loss (pregnancy loss or baby loss)?

Given the diverse ways in which loss and relationship to loss are described, a secondary aim was to examine the language used within the included papers.

## Methods

This qualitative evidence synthesis followed systematic methods throughout the process, including searching, study selection, extraction, analysis and interpretation, and reporting. ENTREQ reporting guidelines were followed (16).

### Search methods for identification of studies

The review question was developed using the PEOS framework, where the population (P) was LGBTQ+ people, the exposure (E) was perinatal loss, the outcome (O) was experiences, and the study design (S) was qualitative research. As shown in **Table 1**, search terms were developed for the two concepts relating to population and exposure (using expansive terms to enable identification of literature that may address experiences of perinatal loss, but without such language, i.e. terms relating to other types of reproductive loss, such as abortion or unsuccessful assisted conception procedures) and adapted for the databases being searched (i.e. PsycINFO, MEDLINE, CINAHL) using a combination of subject headings and keywords. Full text searches were carried out on each database using Boolean phrasing of OR between each row item.

**Table 1:** Search terms.

Population LGBTQ+	Exposure Perinatal loss
LGB* sexual minorit* gender minorit* transgender transsexual transmasculine ‘trans men’ non-binary nonbinary gender divers* lesbian* same gender same-gender same sex same-sex gay bisexual* ‘sexual orientation’	stillbirth neonatal death ‘perinatal loss’ pregnancy loss ‘baby loss’ miscarriage abortion ‘termination of pregnancy’ ‘pregnancy termination’ ‘termination for medical reason’, ‘termination of pregnancy for fetal anomaly’ fetal anomaly maternal bereavement paternal bereavement parental bereavement perinatal death donor conce* adj3 unsuccessful artificial insemination adj3 unsuccessful IUI adj3 unsuccessful ICSI adj3 unsuccessful IVF adj3 unsuccessful donor conce* adj3 fail* artificial insemination adj3 fail* IUI adj3 fail* ICSI adj3 fail* IVF adj3 fail*

Alongside database searching, complimentary strategies were used, specifically forward and back citation chaining (i.e., respectively, checking reference lists within included studies, and checking subsequent studies that cited the included studies) and searches on key authors. Initial searches were conducted in 2021, with updated searches run in November 2024.

### Study selection and eligibility criteria

The screening and selection of relevant articles followed a stepped process according to PRISMA guidelines. Duplicate records and non-English records were initially filtered out via the database filtering tools. Search results were then imported into EndNote Online. Records were independently assessed for eligibility by reviewers (ZD, ABe, MG) using the pre-defined eligibility criteria presented in **Table 2**, using a multi-stage process, i.e. initially based on the titles and abstracts, then the full-text articles. For any records where title and abstract details did not provide adequate information to enable assessment, full-text articles were obtained and assessed by the team to determine inclusion. The screening process was recorded using Word documents linked to EndNote libraries, including documenting reasons for exclusion of full-text articles.

**Table 2:** Eligibility criteria.

Characteristic	Inclusion criteria	Exclusion
Population	Any individual identified in the study as LGBTQ+, regardless of whether they or their partner had been pregnant, and regardless of the length of time since the loss occurred	Articles where the experiences of LGBTQ+ people are not easily distinguished (i.e. research including heterogeneous samples where data relating to LGBTQ+ people and cisheterosexual people are not easily distinguished)
Exposure	Perinatal loss including any loss occurring during pregnancy (i.e. miscarriage, termination, stillbirth) or birth (intrapartum stillbirth) or the first 28 days (i.e. neonatal death); unrestricted by the circumstances relating to the loss (e.g. termination of pregnancy)	
Outcomes	Experiences	
Study design	Empirical qualitative studies of any type and empirical mixed methodology studies where qualitative aspects can easily be extracted	Empirical quantitative research or quantitative components of mixed-methods research; surveys that do not provide qualitatively oriented analysis of open-ended comments; reviews
Geographical location	Any (unrestricted by geographical, legal and sociocultural context)	
Language	English	
Publication date	Any	
Publication types	Journal publication	

### Quality appraisal

The Critical Appraisal Skills Programme (CASP) Qualitative Research Checklist as used to assess the methodological quality of included studies against 10 indicators across various domains, including aims, design, sampling, data collection methods, data analysis methods, interpretation, findings and value of the research (17). Quality appraisal is integrated in the overview of included studies and was used to assess the strengths and weaknesses of the included studies rather than to determine eligibility for being included in the review.

### Data extraction and thematic synthesis

Information about study design, methodology, and participant characteristics were extracted and tabulated (ZD, ABe, MG). Qualitative data from the included articles were synthesised by the full team (ZD, ABa, ABe, MG) using thematic synthesis, as outlined by Thomas and Harden (18). This included extracting text of all findings sections (i.e. treating both participant quotations and author interpretations as data) and then coding each line according to its meaning and content. The participant identifiers used here (numbers and pseudonyms) are taken directly from the included studies; all data is anonymized. Consistent with Thomas and Harden, codes were initially organised into descriptive themes and then further developed into analytical themes, refining through a series of meetings where authors interrogated the developing analytical themes (18). This included considering relationships between themes, supported by visual mapping, and discussing alternative interpretations, before agreeing a final version of the synthesis, reported here.

### Reflexive statement

This review was conducted by a multidisciplinary team comprising academic researchers (Psychology, Midwifery, applied health research) from the Global North, some of whom are also birth workers (doula and midwife), are LGBTQ+, and have direct lived experience of perinatal loss. We recognise that our positionalities shaped the review process, from the commitment to this area and framing of the research question, through to the interpretation of findings. We valued ongoing reflexive dialogue throughout the review, acknowledging how our perspectives influenced theme development and language use. This collaborative and reflexive approach aimed to enhance the rigour, relevance, and sensitivity of the synthesis.

## Results

### Overview of included studies, with consideration of language used, and quality appraisal

**Figure 1:**
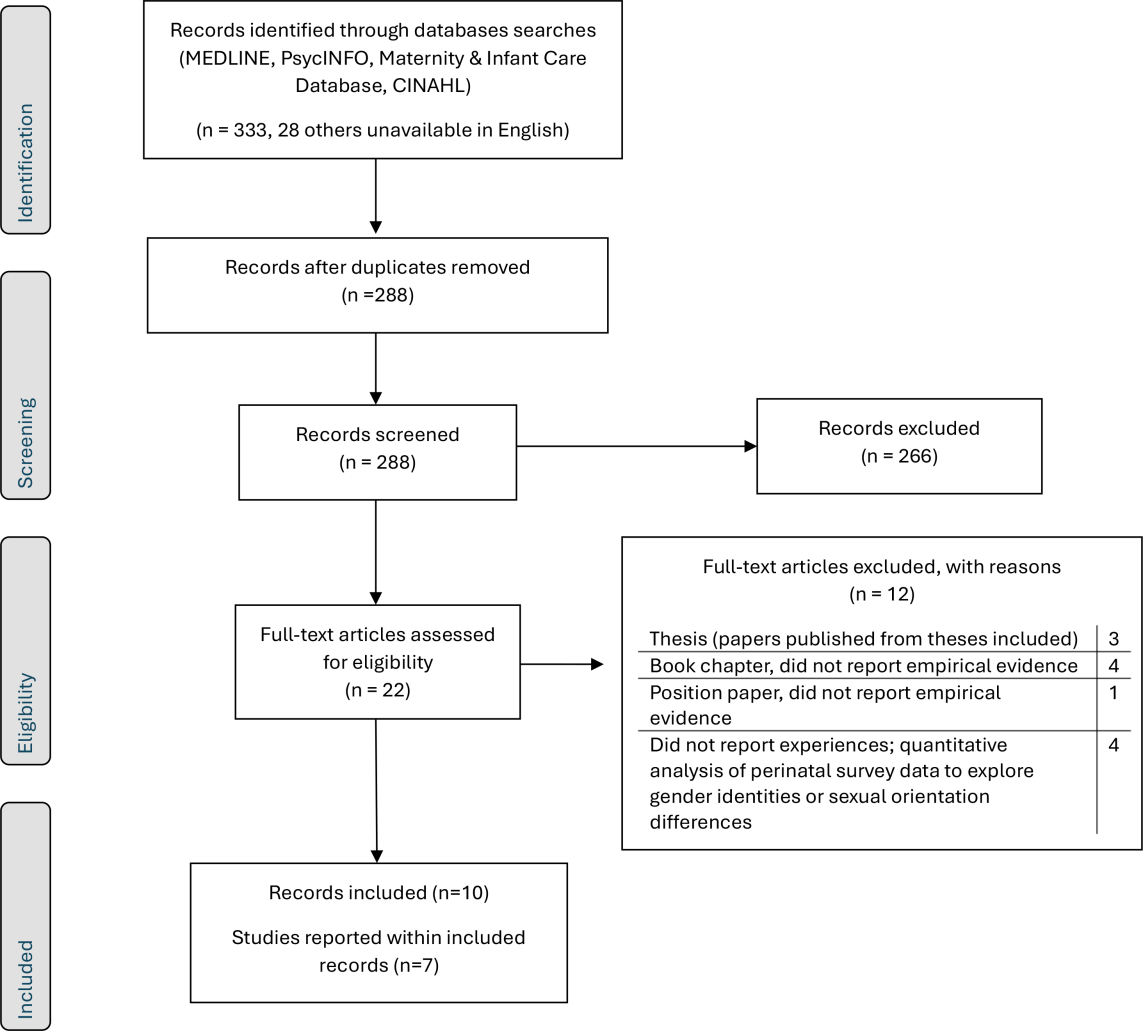
PRISMA flowchart of review process.

As shown in **Figure 1** (PRISMA flow diagram), seven studies met eligibility for inclusion and these were reported across 10 papers. **Supplementary File 1** provides further detail on the studies that were excluded at full text. Details of participant characteristics are given in **Table 3**, with further details of the included studies reported in **Supplementary File 2**.

**Table 3:** Participant demographics in included studies (n=7, reported in 10 papers).

Study ID, first author, year, country, Aim	Sample characteristics (reflecting language used in papers)
1. Andalibi, 2021, USA – reported across 3 papers: a. Pyle, 2021, USA (19). Aim: to investigate (non)disclosure of pregnancy loss among LGBTQ people to known ties on social media b. Lacombe-Duncan, 2022 (20). Aim: informed by minority stress theory, to explore the experiences of multi-level stigma and resilience among LGBTQ+ people in the context of conception, pregnancy, and loss c. Andalibi, 2022 (21). Aim: informed by an intracategorical intersectional lens, to uncover the benefits and challenges of LGBTQ-specific and non-LGBTQ-specific pregnancy and loss-related online spaces	*Sample overview:* 17 LGBTQ+ people *Relationship to loss:* 14 “physically experienced pregnancy loss”, 3 “in an intimate partnership in which pregnancy loss occurred” *Gender:* 15 cisgender women, 1 transmasculine person, 1 non-binary person *Sexual orientation:* 3 lesbian, 1 bisexual, 5 Queer, 2 asexual, 6 multiple orientations *Race/Ethnicity:* Race: 13 White, 1 Black/African American, 1 Latinx, 1 multiple races/ethnicities, 1 human Ethnicity: 1 African American, 7 White, 2 American, 1 Jewish, 2 Latinx, 3 European, 1 multiple *Country:* All USA *Age:* Mean 34.4 yrs (SD 3.3) *Relationship details:* 16 married, 1 single *Loss details* -single losses reported - pregnancy loss (defined as an undesired loss of a pregnancy at any gestational age) in last 2 years - gestation at loss n/r *Conception details:* n/r *Parity:* n/r *Other notes:* Information on education, income, religion, geography, social media use available in papers
2. Cacciatore 2011, USA (22). Aim: to explore child death in same-gendered-parent families; specifically ‘maternally bereaved lesbians’.	*Sample overview:* 6 lesbian mothers *Relationship to loss:* Not specified – describes as maternally bereaved lesbians *Gender:* Language of women is used throughout, without any mention of cisgender/transgender *Sexual orientation:* All lesbian *Race/Ethnicity:* Ethnicity: 4 Caucasian, 1 Jewish, 1 Italian-Irish, 1 European-Canadian *Country:* *USA* *Age:* 3 aged 36-45, 3 aged 46-55 *Relationship details:* 4 partnered (including 1 married in Canada), 2 single *Loss details* - 5 “of the children had died during the perinatal period or birth”, 1 had died in adulthood; describes “all the deaths were traumatic and unexpected” -details of any earlier losses n/r -time since loss n/r *Conception details:* - n/r *Parity:* - inclusive of losses, 2 had 1 child, 3 had 2 children, 2 had 3 children; 2 had no living children at the time of participation Other notes: Information on religion and socioeconomic demographics available in paper
3. Peel, 2010, UK, USA, Canada and Australia (23). Aim: to explore lesbian and bisexual women’s experiences of miscarriage, stillbirth and neonatal death.	*Sample overview:* 60 women *Relationship to loss:* 78% had “physically experienced their loss(es) (i.e. carried the pregnancy)” (also referred to as “birth mothers”), 22% “had experienced loss as the social mother (i.e. the partner of the women who had carried the pregnancy)” *Gender:* Language of women is used throughout, without any mention of cisgender/transgender *Sexual orientation:* All described as non-heterosexual; 76.6% were lesbian, 15% bisexual, 8.3% ‘other’ *Race/Ethnicity:* 92% white (further details n/r) *Country:* 43.3% UK, 28.3% USA, 18.3% Canada, 10.0% Australia *Age:* Mean 35, range 22–55 years *Relationship details:* 82% in relationship with a woman, 8% single, 5% in polyamorous relationships, 3% married to a man, 1 in a relationship with a trans man. (Note: this is current relationship; 90% were in same relationship at time of loss) *Loss details:* - one-third had experienced multiple losses - majority early miscarriage, some late miscarriage, stillbirth or neonatal death - majority experienced most recent loss occurred in past 5 years (83%), including 45% of sample in past year *Conception details:* - all except one were planned pregnancies - 84% conceived using donor sperm (majority at a clinic, some at home, minority via IVF); 14% via sexual intercourse with male partner, 2% via sexual intercourse with a man who was not their partner - conceived within 1 month-10 years of trying (mean 9.2 months, SD n/r) *Parity:* -unclear: reports that 55% had children, with mean age 4.5 years (range 4 days-17 years); also reports number of losses Other notes: Information on social class and disability available in paper
4. Riggs, 2020, Australia, USA and European Union (24). Aim: to explore experiences of pregnancy loss among a sample of men, trans/masculine, and non-binary people who had undertaken a pregnancy	*Sample overview:* 16 men, trans/masculine, and non-binary people *Relationship to loss:* All had experienced pregnancy loss, “having undertaken at least one pregnancy following a gender transition” *Gender:* Collectively described as men, trans/masculine and non-binary; multiple genders listed; most used he/him or they/them pronouns *Sexual orientation:* Range of sexual orientations (e.g. bisexual, fluid, gay, human-sexual, pansexual, queer), including multiple stated; 2 n/r; 0 stated heterosexual *Race/Ethnicity:* n/r *Country:* 3 Australia, 8 European Union (including UK), 5 US *Age:* Mean 35, range 23-49 *Relationship details:* 2 single/no partner, 1 casual partners, 13 in a relationship (including 1 now separated; 4 with cisgender woman, 6 with cisgender man, 3 with transgender man) *Loss details* -10 had one pregnancy loss, 6 had multiple - Gestational age at loss ranged from 3–23 weeks; authors described as 19 early term losses (12 weeks or less) and 5 late term losses. -Time since loss n/r *Conception details:* - n/r *Parity:* Separate to the pregnancy losses, 13 had live births (range 1-4) and the remaining 3 were pregnant at time of interview Other notes: Taken from a wider trans masculine pregnancy project involving 51 participants; 16 of whom had experienced loss
5. Rose, 2022, 2023, Australia and England – reported across 2 papers: a. Rose & Oxlad, 2022 (25). Aim: to explore how the context in the lead up to loss was important for LGBTQ+ people when pregnancy loss occurred b. Rose & Oxlad, 2023 (26). Aim: to explore LGBTQ+ people’s workplace leave and support experiences following pregnancy losses as gestational or non-gestational parents	*Sample overview:* 14 LGBTQ+ people *Relationship to loss:* - 5 experienced pregnancy loss as gestational parents (only) - 4 experienced pregnancy loss as both gestational parents and as non-gestational parents (where a partner was pregnant) -5 cisgender men experienced pregnancy loss as non-gestational parents in context of surrogacy *Gender:* 8 cisgender women, 5 cisgender men, and 1 trans non-binary person; although eligible, 0 intersex or asexual people took part *Sexual orientation:* Reported as a range represented within LGBTQ+; 11 were in a same-gender relationship at the time of loss *Race/Ethnicity/’Cultural diversity’:* -includes 2 Aboriginal and Torres Strait Islander, 1 Greek-Australian (thesis reports remainder of cultural identities as: 2 n/r, 1 Irish, 1 4^th^ generation Australian, 1 English, 1 Anglo-Australian, 2 Anglo-Celtic, 3 White; with the 2 Aboriginal and Torres Strait Islander self-describing as Caucasian/Indigenous and Aboriginal-mixed) -2 had partners for whom English was a 2^nd^ language *Country:* Australia (12) and England (2) *Age:* 30–60 years (mean 40) *Relationship details:* All in long-term relationships *Loss details* - 2 experienced 1 pregnancy loss; 12 experienced multiple (2–5) - details on gestational age at loss n/r; includes “ectopic pregnancies, miscarriage, medically induced termination and stillbirth” -notes that some also experienced “other reproductive losses, including unsuccessful egg insemination or embryo transfers” - time since loss: 8m-10years (mean 3.78yrs, SD 2.59) *Conception details:* - n/r *Parity:* - 6 caring for living children at the time of interview (details n/r; thesis reports 8 have living children) Other notes: Paper 5b is limited to a subset of the 12 participants residing in Australia. Information on surrogacy details available in papers. Further sample details available in thesis (including religion).
6. Røseth, 2023, Denmark and Norway (27). Aim: to explore how Danish and Norwegian lesbian couples bereaved of a child in the perinatal period experience the loss, and to examine the maternity care offered	*Sample overview:* 6 lesbian couples *Relationship to loss:* 6 birth mothers/biological mothers and 6 non-birth mothers/co-mothers *Gender:* Language of women is used throughout, without any mention of cisgender/transgender *Sexual orientation:* All lesbian *Race/Ethnicity:* n/r *Country:* 4 couples Danish nationality and living in Denmark, 2 couples Norwegian nationality and living in Norway *Age:* 29–42 years *Relationship details:* “Living in a relationship with another woman when they lost their baby” *Loss details* - gestation at loss 21–40 weeks (eligibility was “late pregnancy or the first few days after birth” and some included birthing a baby who “had already died or risked dying”) - losses occurred within last 3 years *Conception details:* - n/r *Parity:* - n/r Other notes: None
7. Wojnar, 2007, USA and Canada (28). Aim: explore miscarriage amongst lesbian couples	*Sample overview:* 10 lesbian couples *Relationship to loss:* 10 ‘birth mothers’(“the partner who conceived and miscarried pregnancy”) and 10 ‘social mothers’ (“the birth mother’s female partner”) *Gender:* Language of women is used throughout, without any mention of cisgender/transgender *Sexual orientation:* All lesbian *Race/Ethnicity:* All white *Country:* USA and Canada *Age:* 30-45 (birth mothers: mean 37.6, SD 4.19, range 30-42; social mothers: mean 38.0, SD 4.60, range 31-45) *Relationship details:* In ‘committed’ relationship (at time of loss and still in that relationship) *Loss details* - 1–4 miscarriages - gestation at loss 5–20 weeks, mean 11.0 weeks, SD 4.34 -losses occurred 1 week-2 years prior to study enrolment, mean 11.6 weeks, SD 4.25 *Conception details:* - 5 used ‘alternative insemination’ with known donor, 5 used unknown donor; n/r whether clinic or home *Parity:* - 0–3 living children: previous to loss, 5 birth mothers had given birth,0 social mothers had given birth Other notes: Information on education and employment available in paper

Language used in the sample characteristics reflects the language used in the papers (e.g. use of people, parent, mother).

All were focused on pregnancy loss and/or baby loss; none were focused on earlier reproductive loss (such as assisted conception attempts that did not result in pregnancy), although study 5 noted these in addition to the perinatal losses being described. Loss-related language differed within the study aims, with three focused on pregnancy loss (studies 1,4,5), two on child bereavement or child death in the perinatal period (studies 2,6), and two using language referring to specific clinical circumstances (study 7: miscarriage, study 3: miscarriage, stillbirth and neonatal death). All were conducted in the Global North, spanning North America (studies 1,2,3,4,7), Europe (studies 3,4,5,6), and Australia (studies 3,4,5). Study 3 collected data via (online) survey methods and acknowledged this as a limitation in precluding ability to probe further. All other studies used semi-structured interviews: individual interviews (studies 1,2,4,5), couple interviews (study 6) or both (study 7); some in-person only (studies 2,7), some remote only (studies 1,4) and some using combinations (studies 5,6).

Three studies – which consistently used language that framed the participants as parents – were focused on lesbian mothers, either as individuals (study 2, unspecified whether these were gestational parents) or as couples (described in study 6 as birth mothers and biological mothers, and non-birth mothers and co-mothers, and in study 7 as birth mothers and social mothers). One study (study 3) focused on pregnancy loss in “lesbian and bisexual women”, using the terms “respondents” and “women” in the findings, but distinguishing in the aims and methods between participants who were “birth mothers” – described as those who had “physically experienced their loss(es) (i.e. carried the pregnancy)” - and “social mothers” or “non-birth mothers” as “the partner of the women who carried the pregnancy”. None of the four studies that were focused on lesbian mothers or on women specified whether participants were cisgender.

Only one study was focused on trans men and non-binary people (study 4). All participants were identified as people who had “undertaken at least one pregnancy following a gender transition”; the sample was not described as parents and the findings noted variation in whether participants spoke about pregnancy losses “as children”, with the term “gestational parent” reserved for the discussion section.

The two studies that were framed around “LGBTQ(+) people” (study 1 and study 5) both explicitly included cisgender and transgender participants. Study 1 distinguished in the methods between those who had “physically experienced pregnancy loss” and those “partnered with someone who physically experienced pregnancy loss”, also referred to in 1b as “non-gestational parents”. Study 5 distinguished in the methods between gestational and non-gestational parents. Both studies predominantly used the language of participants or people within the findings, with exceptions relating to “parenthood” or “becoming parents”.

Study 5 was the only study to report some participants having experienced perinatal loss both in gestational and non-gestational roles, and was also the only study to include perinatal loss experiences in the context of surrogacy.

Reporting of demographics characteristics was not consistent across the included studies. Diversity concerning race and/or ethnicity reporting was variable across studies, being evident in studies 1 and 5, and to a lesser extent 2, but minimal or absent in others (respectively 3 and 7), and unreported in 4 or 6. Some studies reported figures for socio-economic indicators including education (studies 1,2,3,7), religion (studies 1,2,5), and disability (study 7) sometimes noting a lack of diversity in respect of these characteristics as a limitation. Participant demographics are available in **Table 3**.

All studies provided clear statement of aims and used methodologies and research designs that were appropriate to address the stated aims. Recruitment strategies generally relied on use of personal or research networks, social media and community organisations; mostly LGBTQ+ focused whilst some were specific to pregnancy or perinatal loss. Methods of data collection (including modality and duration) were provided for most studies and were appropriate (study 2 did not report whether these were in-person, which may reflect the study’s age). Ethical issues were considered appropriately. Data analysis was generally rigorous and built high trustworthiness, with detail provided to offer transparency of the analysis process, researcher reflexivity (which may include addressing direct personal experience (e.g. study 1) or collaborating with experts with lived experience (e.g. studies 2,4), use of member checking (e.g. studies 2,5,7), peer debriefing/discussion (e.g. studies 1,2,4,7). Such aspects were not documented in study 3, which was the only study to involve analysis of open-ended survey data. None of the papers provided **Supplementary Materials** to show development of themes however it is noted that such aspects can be impacted by journal restrictions on length and changes to reporting practices over time. All studies provided explicit findings and identified the distinct contributions made, together with implications for further research.

### Thematic synthesis

Thematic synthesis of the seven included studies generated one overarching theme - *enduring hope and loss* - and three connected themes: 1. Investment, which included the effort of navigating cisheteronormative systems, frequently after investing time, finances and emotions in assisted conception processes; 2. Support in relation to loss, especially while being marginalised, excluded, or feeling invisible and even unsafe as an LGBTQ+ family, and 3. Meaning-making during the immediate experience, in the aftermath and beyond, and as queer bodies.

Illustrative quotes from participants are indicated by the use of their identifier (where reported in the included study); illustrative quotes from the interpretation by the authors of included studies are indicated by ‘author interpretation’.

#### Overarching theme: enduring hope and loss

**Figure 2:**
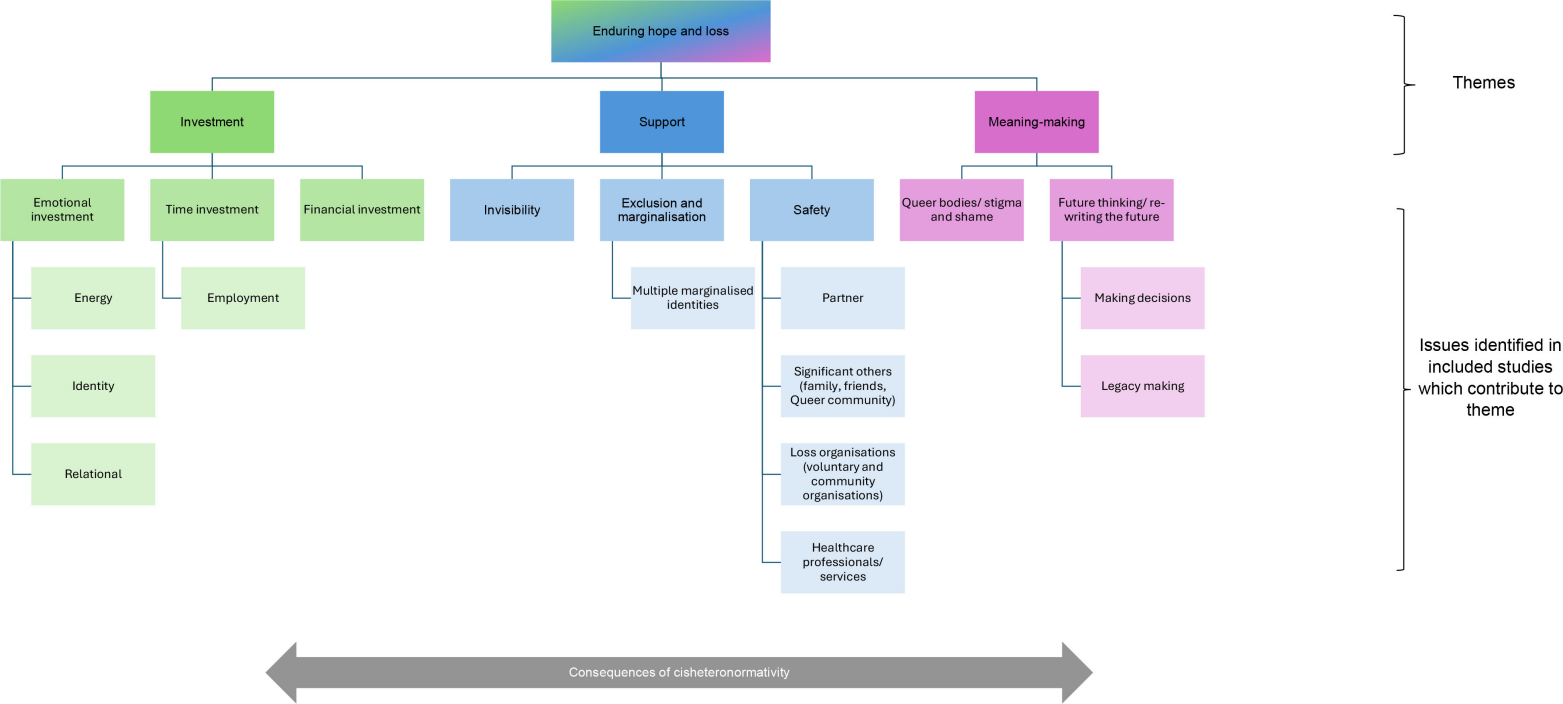
Thematic synthesis map.

As depicted in **Figure 2**, the three main themes we generated sat within an overarching container of *‘enduring hope and loss’*. This overarching theme reflected the complexity of perinatal loss, its multiple layers, and, for most of the participants, the profound emotional pain and distress it brought – for some, pain and distress that persisted over a long period. *Enduring hope and loss* connected with the other three themes in multiple ways. Firstly, experiences are described as being impacted by the protracted time involved in becoming pregnant as LGBTQ+ people, typically involving additional processes and considerations, including those relating to assisted conception. This was expanded upon in Theme 1 and resonated with the included studies’ ‘amplification of loss’ (study 3) and ‘compounded by complexities of planning’ (study 7). Here, the endurance spoke to losses typically not being experienced as isolated incidents or time-specific. Secondly, such experiences could be compounded by enduring these without visibility or access to support, as expanded upon in Theme 2. For some, this occurred without invisibility as people who were pregnant, or whose partner was pregnant, or whose surrogate was pregnant; for some, this is without being visible as parents, or without their babies being visible as babies. Thirdly, for some, the enduring loss was accompanied by enduring hope and strength; this was explicitly described as resilience in study 1b. This enduring hope persisted when pursuing desires for pregnancy and parenthood, often whilst needing to overcome considerable adversity to become pregnant, or other losses that were separate to the perinatal loss itself. These experiences were closely tied to processes of coping and meaning-making, which are further explored in Theme 3.

#### Theme 1 investment

For many LGBTQ+ people, perinatal loss represented not only the loss of their imagined future but also the loss of significant investments of emotions, time and finances in becoming pregnant.

*“The intentionality and the effort that goes into the process of getting pregnant is different…. It just sort of feels like we’ve had to really fight for this.” (P7, 30s, White, Queer/Lesbian/Gay, Cisgender) (*20)

*“…. The stakes of miscarriage for queer people can be really, really different … I just know so many queer people who have had a really hard time getting pregnant, and it’s not something that I feel like we, as in like people in kind of like queer communities, talk about openly. I think there is a lot of isolation often in the process…. I think straight people just can’t wrap their head around what it often takes for queer people to create families” (P10, 30s, White, Queer/Lesbian, Cisgender)” (*19)

This was described as intertwined with the grief and compounding it further:

‘the experiences of miscarriage and conception for lesbian couples were so intertwined that one cannot fully comprehend lesbians’ experience of miscarrying without understanding their difficulties conceiving’ (author interpretation) (28)

‘While most people who experience pregnancy loss share experiences of grief, this grief may be amplified and compounded for LGBTQ people, largely because of the increased effort involved with planning and achieving pregnancy.’ (author interpretation) (19)

For most LGBTQ+ people planning a pregnancy, a considerable amount of emotional work took place before conception was attempted. In choosing to pursue pregnancy, LGBTQ+ participants in the included studies reported that they had to consider multiple questions about how to try to conceive. Questions included who was going to carry the pregnancy and whose gametes would be used. Where additional gametes were required, questions included: whether to use known or unknown donors, which donor to select, what method of conception would be attempted, and when to attempt conception.

*“I wish I could communicate to people how invested we were, we are … so it’s not just about a pregnancy. It’s about thousands of hours of research. Thousands of hours of looking out for ourselves, and just that leap of faith!” (David) (*25)

Where the person trying to conceive had a partner (or partners), discussing these decisions had included a significant amount of relational work, prior to attempting conception. Evident in the included studies were examples of the strengths accompanying this relational work.

‘The decision to pursue parenthood … involved several processes: self-reflection about whether it was right to pursue motherhood, reconciling their differences, locating a donor, finding a nonhomophobic provider, and … [was] often delayed by differences in partners’ readiness for pregnancy and fears of raising a child in a society that discriminates against nontraditional families.’ (author interpretation) (28)

For some trans men, non-binary folk and masculine lesbians, accessing care also involved being misgendered by fertility or perinatal services, adding to the burden placed on people:

*“… my wife had to navigate as a masculine woman going through the trying to conceive process of accessing care, going to a clinic and being misgendered.” (P14 Latinx, Mexican and Salvadoran, female, queer, married, 50–75K) (*20)

Some trans men spoke of the risks involved when accessing medical help during a perinatal loss, including implications for their safety when ‘outed’ as transgender by requiring such care:

*“[attending a hospital during a miscarriage] was a big decision, considering I was going to have to out myself to a hospital full of strangers. I have never had to go to the emergency room as a trans guy before and it’s always been a huge fear of mine”(Trent) (*24)

The emotional investment that accompanied decision-making was additionally accompanied by significant investment of time:

*“it has been a very long and distressing journey of over 8 years … waiting for appointments with clinics, waiting to hear the answer from their ethics committee, waiting for a friend to say yes [to donating his sperm], waiting for ovulation, waiting for a period, waiting… ‘ (R8, Australia).” (*23)

This included investment relating to the practicalities of attempting to conceive, regardless of whether conception was attempted at home or in a clinic:

*“when lesbians get as far as getting an appointment with the GP [family physician] to be referred to a clinic or decide that they are definitely going to try to find a donor, they should get a congratulations card!! It’s the equivalent of many heterosexuals giving birth or at least getting pregnant! It’s usually the result of years of talking and planning and working out how to do it. (R41, UK)” (*23)

Where this required leave from work, revealing the reason meant individuals had to disclose that they were trying to conceive:

*“[I had] to explain to my work, why we needed some time off work. I was forced to tell people that I didn’t want to tell” (James) (*26)

Taking time off could be complicated not only for those who were pursuing or had been physically pregnant, but also for their partners and people using surrogates: this often meant not only disclosing pursuing parenthood, or indeed being expectant parents, but also potentially ‘outing’ themselves in relation to gender and/or sexuality and/or routes to parenthood, each of which could carry additional stigma:

*“[If you tell] people … we were having babies through surrogacy and [the babies] died … people immediately jump to judgement” (James) (*26)

In cases where perinatal loss and fertility treatments had to be taken as sick leave, or where LGBTQ+ people chose to take sick leave to avoid disclosure, the amount of time people felt able to spend recovering from a loss was sometimes compromised:

*“I felt this really strong pull to not have days off work because I knew I needed the sick days for our next treatments” (Cate) (*26)

Financial investment relating to conception was identified across the included studies. These ranged from paying travel expenses or fees to a known donor, to clinic fees for intrauterine insemination (IUI) or *in vitro* fertilisation (IVF), to paying expenses or fees to surrogates. Such costs could also be accompanied by feels of resentment, anger and injustice:

*“I have this deep constant anger that IVF is one of the only ways same-sex couples can start a family. The effort involved, not to mention the exorbitant cost.” (Cate) (*25)

Financial investments and time investments were sometimes traded against each other – for example it could be cheaper to use a fertility clinic abroad, but this might require more significant periods of time away from work, or from older children. These decisions were also affected by the legal situation in the individual’s country. Additionally - as noted in study 5 – some experienced their loss in the context of a series of other reproductive losses, including cycles of conception attempts that did not result in pregnancy but nonetheless carried emotional, time and financial investments.

For some LGBTQ+ people, the investment required to achieve a pregnancy meant that the loss of the pregnancy was also the loss of their chance at biological parenthood:

*“loss of an option” (Ben) (*24)

*“Our surrogate was pregnant eight weeks with twins, and then Cambodia overnight banned surrogacy … if she lost it, if she miscarried, that’s it for us, that is really it for us, because we can’t move these embryos to another country.” (David) (*25)

Coming to terms with these multiple additional losses added complexity to the experiences of grief. These experiences, although common with cisgender heterosexual people utilising assisted conception, additionally required individuals and couples to navigate cisheteronormative services and systems during their bereavement:

*“There was no place to put [my partner’s] information on the death certificate … and, well, that’s just not right.”(Participant quote) (*22)

*“my partner was asked to leave during several exams, and was not allowed to answer questions regarding the autopsy or funeral arrangements after stillbirth’, R46, USA” (*23)

Cisheteronormative norms often meant that LGBTQ+ people’s perinatal losses, their relationships and families were simply not recognised; nor were their accompanying emotional and practical investments. This lack of recognition also sometimes meant being unable to access resources that would be available to cisheterosexual couples, requiring further investment while dealing with perinatal loss:

*“They [HR] wouldn’t give it [leave from work] me. She said “You were never pregnant; your partner was never pregnant. You can’t have it.” I had kids in intensive care in India … And she [HR] wrote to [government department] and tried to get a ruling to say that we didn’t get to have leave, because they weren’t our children.” (James) (*26)

*“We told [the midwife] we were using my wife’s eggs and we’d had an ectopic pregnancy before, she kept on saying ‘Oh, but those weren’t your pregnancies. They were your wife’s pregnancies, because they were your wife’s eggs.’ And I explained ‘No, but I carried’. I had the miscarriage. My wife also lost a potential child in that situation, but it’s very invalidating to say that that wasn’t my pregnancy because it was someone else’s egg. And I know that they wouldn’t have said that if I’d had IVF and used donor eggs for another reason.” (Elizabeth) (*25)

#### Theme 2 support

The included studies showed that LGBTQ+ people looked for support from their friends, families, and from formal perinatal loss services, but their experiences of perinatal loss often remained invisible to those around them, or required additional work to make them visible. Some voiced invisibility as linked to an assumption that LGBTQ+ people will not attempt to become biological parents.

*“My sister did say something like, ‘Well, you know, why are you guys even trying to do biological kids? Why not just adopt?’ And I was pretty offended. I was like, ‘Why didn’t you?’ People can choose to have biological children regardless of their sexual orientation … So, I felt like there was this different standard for me, and that I shouldn’t even want this…” (P15, 30, Partner, White, Lesbian, Cisgender)” (*19)

Invisibility led to the unintentional exclusion of LGBTQ+ people from support that would have been available to cisgender heterosexual people experiencing a perinatal loss. In LGBTQ+ communities and friendship groups, whilst parenthood is becoming more common, many people will not have children. For some, perinatal loss was therefore a subject which was less easy to discuss, and one for which there was less community experience of supporting:

*“I was surprised to encounter heterosexuals who were more supportive of us than some members of our lesbian community.”(Participant quote) (*28)

Wider family were sometimes surprised to learn that perinatal loss had occurred, where they were unaware that their LGBTQ+ family member had been attempting to conceive. Some LGBTQ+ people spoke of not disclosing their loss to family or friends to protect themselves from discrimination when grieving. This was sometimes linked to others’ known or anticipated belief that raising children in anything other than a cisheterosexual couple was immoral or damaging to the child, and some individuals faced this kind of discrimination whilst grieving their loss.

*“This is very painful to talk about, but my family is not supportive of our relationship, so I didn’t tell them because I knew they would never express sympathy.”(Participant quote) (*28)

*“I don’t want people coming after me and telling me, “Well, maybe this loss happened for a reason because you shouldn’t be trying to have a child in the first place.” (P7, 30s, White, Queer/Lesbian/Gay, Cisgender) (*20)

Where LGBTQ+ people accessed support services specifically for perinatal loss, some found them to be cisheterocentric, which caused further distress:

*“[whilst experiencing a loss, they were asking] ‘so like, they have the same father? So, they have different donors. And [I had to] explain things like that. So … educate them … explain what it’s really like.” (Else and Siri) (*27)

Anticipating this, some who accessed support organisations for perinatal loss chose not to disclose their LGBTQ+ identity, which was sometimes accompanied by complicated and challenging feelings:

*“I was by myself, and I couldn’t share with them that part of my life … about being a lesbian because the last thing I needed was to be ostracized because of that. I realized that I had censored myself.” (Participant quote) (*22)

Others simply chose not to access support from organisations:

*“I just was not interested in having to deal with homophobia or even just having to explain myself and having to explain what my process had been like or what I had been doing in that space when really, what I would have needed out of that space was support and community.”(P9) (*21)

Having to educate people whilst trying to access support was difficult, and on the occasions where LGBTQ+ specific support was available, the ease of access was commented upon:

*“It’s nice just to not have to explain my family structure, or have people assume pronouns of my spouse or anything like that.” (P7) (*21)

Others reported the importance of receiving inclusive care from healthcare professionals when access care in the context of loss:

*“I had a wonderful doctor who was understanding and took care of me and respected my gender and all that. I needed that in that moment. I needed to not be misgendered on the worst day of my life and I had that.” (P3, 30s, White, Queer/Bi/Pan, Nonbinary)” (*19)

*“‘the doctors and nurses were great—no homophobia, no problem at all with us. They automatically gave my girlfriend the consent form to sign (or whatever it was—I don’t know)—they just treated her as my partner, no questions or issues which was a huge blessing in those circumstances’ (R41, UK).” (*23)

However, for some LGBTQ+ people, their experiences and existence were marginalised, minimised or dismissed. This included by not being seen as grieving parents, or by the adults and existing or future children not being seen as a family. Evident within two of the included studies were examples of the importance – where applicable - of both people being addressed as parents.

*“Some health professionals seemed unable to understand my partner’s distress at losing her child … I don’t think they understood what it meant for my partner, that she was a parent and she had lost her baby too.” (R45, UK) (* 23 *).*

‘Thus, being recognized as the baby’s parents was important, particularly for co-mothers, even if this meant being a parent of a dead child. Midwives’ active use of the word “parents” strengthened the couples’ understanding that they were in fact parents,

and in hindsight, they described how memories of these situations activated feelings of

tenderness and grief. … [and] helped to develop a couple’s parental identity.’ (author interpretation) (27)

Some LGBTQ+ people also experienced messages that the potential future child was not perceived as a child of value by the adults’ support systems or by society in general (study 2).

*“People have expressed their belief that we will psychologically damage our children simply because we are gay … therefore … my child who died would be looked upon as “less than” the children lost by heterosexual couples, that my child would not hold the same “value” as a human being to others because she was born to a lesbian mother.” (Participant quote) (*22)

Study 1 found a lack of representation in online spaces for LGBTQ+ people of colour. Similarly, accessing appropriate in-person support was more difficult for people with multiple marginalised identities:

*“And those had become some of my supports that I also was feeling very othered. I don’t identify as a heterosexual person, I actually identify as a bisexual person and at the time my spouse was identifying as a woman and my spouse is now trans. So just like kind of going to some of these, the in-person groups and even being online and just seeing that it was very like cis-het groups, very cis-gender, heterosexual groups, everybody’s talking about mommy and daddy, everybody’s talking about that kind of thing. And in addition to that, also feeling very othered ‘cause we were like the only black people in any of these groups most of the time … It just felt very uncomfortable, the language that they were using.” (P13) (*21)

Indeed, a participant from that study had created a group for other Black people whilst navigating their own grief.

#### Theme 3 meaning-making

The included studies illustrated examples of people trying to make sense of their perinatal loss through creating meaning from what happened across the immediate experience of loss, the aftermath of the loss and the care received, and the time beyond. Meaning-making was sometimes specific to being a Queer body, and sometimes specific to being in a Queer relationship where future reproductive choices included a different range of options about routes to pregnancy than cisheterosexual people would have.

In the immediate experience and the aftermath, some LGBTQ+ people who had carried the pregnancy experienced complex emotions about their bodies. For some, there was a sense of ‘double failure’, as shame from internalised homophobia or transphobia interacted with shame related to perinatal loss. For others, the relief of being able to become pregnant affected their experience of grieving the perinatal loss.

*“It felt like, [the pregnancy loss] felt more positive than negative. It was sad, but more ‘like this can happen’.” (Will) (*24)

For partners of people who physically experienced the loss, their feelings could be complicated:

*“[talking to partner] you’ve used your body for all this, while I just have to stand on the sidelines.” (Signe and Trude) (*27)

There were examples of people in partner roles feeling particularly unsure about their role and expectations in the context of perinatal loss, as they tried to navigate their loss both as individuals and as couples. This could be further complicated for LGBTQ+ people who had not been exposed to experiences of others in similar circumstances or with shared identities, relationships or involvement of health services:

*“It is a learning experience for me to know how to be a social mother in this situation [miscarriage] because I don’t have any examples for my role. And I found it paralyzing.”(Participant quote) (*28)

For other LGBTQ+ people, the paucity of legal options available when creating a family became part of how they made sense of their loss:

*“[lack of legal protections in altruistic surrogacy], that’s why we did commercial surrogacy, and let’s jump forward to Hazel [daughter who died]. If that pregnancy were here [in Australia], Hazel would be alive.” (David) (*25)

Findings from included studies were understood as indicating a re-writing of the future that they had begun to imagine, as parents and with hopes for their future child(ren). Making sense of this enforced rewriting included making decisions about whether to try to conceive again or not. For some LGBTQ+ people, making decisions about future attempts to have a child involved additional emotional labour, for example in revisiting questions of who will try to conceive and how they will do so (as discussed in Theme 1):

*“I made the decision … for me to stop. So, the next stage will be for my partner to carry … that was a lot. And I did seek counselling through the fertility clinic in that period because it was two losses and then a decision to stop. You know? It was really hard. It was a tough time.” (Gillian) (*25)

*“[after discovering the donor to their existing child and the baby they lost was a carrier of genetic abnormalities] at that point we’d already decided long ago that W would have another donor. Because none of us dared to do anything, with G’s donor, so like for better or for worse, or because we could go through a pregnancy that was safer for us. And it’s a bit difficult that we’ve got nothing … that looks like G.” (Tine and Marie) (*27)

Additionally, positives were evident, with some participants using the experience of loss to create positive change for the future. Some wished to make sense of the loss by creating a legacy from their experiences. Sometimes, this was achieved by turning the loss into an educational opportunity, to prevent the same experience happening to another parent, or to normalise the experience of loss and grief. Experiencing poor care due to cisheterosexism was one area where LGBTQ+ people who had experienced a perinatal loss wished to focus educational activities to create a legacy from their experiences.

*“I’ve since joined three or four different groups geared towards LGBT and trans people trying to conceive and with babies and breast feeding and I couldn’t find one that was good specifically about loss and miscarriage and I actually started one.” (P3, 30s, White, Queer/Bi/Pan) (*19)

## Discussion

This review found that for many LGBTQ+ people, perinatal loss represents the loss of significant investments of emotions, time and finances in becoming pregnant, as well as the loss itself. Coming to terms with these losses adds complexity to the experiences of grief. These experiences may be further complicated by internalised homophobia or transphobia which surface as LGBTQ+ people try to make sense of the loss, or revisit decisions about the path to parenthood where there are multiple options for carrying a future baby or for sourcing gametes. Following a loss, LGBTQ+ people have similar support needs as cisgender heterosexual people, but support may be less readily available, and where it is available, access may carry costs for LGBTQ+ people relating to psychological and potentially physical safety. These gaps in available accessible support may be compounded by other forms of discrimination for LGBTQ+ people with multiple marginalised identities. Accompanying these layers and complexity of loss experienced by LGBTQ+ people are aspects indicative of strength or resilience; these are collectively captured in the overarching theme – *enduring hope and loss*.

Permeating throughout all themes were the consequences participants experienced arising from interacting with systems designed for cisgender heterosexual parents and the negative consequences of assumptions, both within health systems and societally. Our analysis demonstrates that LGBTQ+ people’s experiences of perinatal loss and resulting needs are actively impacted throughout *by* cisheteronormative pre-conception and post-conception systems, including the (in)visibility of their loss, and their sense of safety in accessing support. Assumptions contributed to the consequent disenfranchisement, which they experienced was evident across studies and explicitly articulated in study 2 when coining the term ‘double-disenfranchisement’ (22). This may hold relevance in understanding the range of language used within the papers and by the participants: individuals and couples experiencing a loss that is not openly recognised, validated or supported vary as to whether they identify with language such as *bereaved parent* and *baby loss*. Indeed, wider LGBTQ+ literature on perinatal mental health identifies the lack of a parental identity template for LGBTQ+ non-birthing parents as a stressor that can precipitate depression and anxiety (29), and our analysis here extends this to the lack of a template for parental identity in the context of LGBTQ+ perinatal loss.

Other consequences of cisgender heterosexual assumptions included how the existing or future potential children of LGBTQ+ parents were viewed by others, and how LGBTQ+ people’s emotions about those views – and sometimes accompanying discrimination - were navigated. These findings show the importance of considering the experiences and needs of LGBTQ+ people experiencing perinatal loss, and the consequences for LGBTQ+ people when services are based upon cisgender heterosexual populations. More broadly, our findings cohere with research that indicates that people’s experiences are affected by the context in which loss takes place (25). For example, assisted conception or third party reproduction featured for the majority of participants in the included studies and aspects cohere with previously documented impacts relating to reproductive trauma, silent grief and lack of support (30, 31).

Lastly this review shows the importance of not conflating role and gender in understanding people’s experiences and needs relating to perinatal loss. Prior to this review, experiences of people in non-gestational roles have been summarised in key reviews on men’s experiences (11, 32, 33). The findings here concerning experiences of people in non-gestational roles (including female partners of pregnant people, and people using surrogates) have parallels with the systematic review and emerging theoretical model offered by Obst and colleagues which identifies community factors (e.g. stigma relating to their grief as men and as people who have not been pregnant) and public policy factors (e.g. lack of bereavement leave for partners) as implicated in men’s grief following perinatal (11). Additionally, one of the included studies found parallels with the experiences of men who physically experienced perinatal loss and the literature on perinatal loss experiences in cisgender men extending the literature on men and perinatal loss (24).

### Implications for services

Our findings are relevant to services who provide support following a perinatal loss. Perinatal loss is an area where many people’s experiences are not visible to or validated by others, including lack of recognition of them as bereaved parents. It is already known that some disparities (such as gestation when the loss occurred) affects the likelihood of help-seeking behaviours and support being accessed (34). Our analysis demonstrates that additional considerations should be given to the likelihood of LGBTQ+ people seeking help and support, as they may feel isolated in their experiences (25), or lack understanding from family and friends (24). The societal invisibility of LGBTQ+ perinatal loss may impact whether people disclose their loss within their social network, due to fears of homophobia and/or transphobia (19). This review also highlights the importance of consideration of intersecting minoritised identities and indicates the need for greater understanding of intersectionality in the context of perinatal loss, as has been noted in research with populations presented as cisgender and heterosexual (35). This matters given the disparities that discrimination brings to duration and quality of healthcare encounters more widely, including aspects such as empathy; as indicated in research concerning racism and racial attitudes (36, 37).Services which aim to provide support to people who have experienced a perinatal loss, or who provide care during subsequent pregnancies should give additional consideration to the needs of LGBTQ+ people, who may both require additional support if they are unable to access support from family and friends, and simultaneously be less likely to seek that support. In line with Aggarwal and Moatti’s conclusion, we recommend that such consideration should include training for staff (38). These implications are relevant for any staff, not only the nurses, midwives and obstetricians who provide perinatal loss care – and this has been highlighted elsewhere in the context of stillbirth and neonatal death, but without consideration of LGBTQ+ aspects. Ensuring that potential LGBTQ+ service users are aware that such training has been undertaken and that a service is safe for them to use without encountering homophobia, transphobia or cis-heterosexism is also necessary.

### Implications for research

Further research is needed if we are to understand the experiences and needs of LGBTQ+ people in pregnancies subsequent to loss. Research with populations that have been assumed to be cisgender and heterosexual finds considerable impacts both for gestational and non-gestational parents, but also that these may differ, with calls for attending to “disparities in the grief process between men and women” (39). A meta-analysis of psychological impacts of perinatal loss found elevated anxiety and depression symptoms in women in pregnancies subsequent to perinatal loss, and identified the need for further research on psychological distress in partners (6). Indicated by the current review is the importance of attending to the experiences and needs of partners of all genders in such future research. With LGBTQ+ people being more likely to conceive using assisted conception, further research is needed to both understand the impact for individuals and for couples of previous perinatal loss on future reproductive choices, with subsequent attempts potentially constrained by depleted financial, time, physical and emotional resources (25), and to investigate the role of wider reproductive losses. Further research to quantify these effects would be useful to understanding LGBTQ+ perinatal journeys. Critical too is that future research be intersectional and for this to feature in team composition, overall design, recruitment and questions asked, through to interpretation. Examples here include the urgent need for research to address the intersection between racism and sexual and gender diversity, and to address gender and sex diversity more expansively.

Transparency of reporting also warrants consideration in future research. As is evident from the included studies, there are significant variations in language regarding parental roles. This includes variation about whether someone who has experienced a perinatal loss is described as a parent and diverse descriptions given to different roles within families (e.g. birth(ing), biological, gestational, non-gestational, social). In individual clinical interactions, it is critical to be individualised with the language used, using the preferred language of those to whom care is provided, which includes recognising a variety of ways in which people will identify themselves and their losses and bereavements. As is evident from the included studies, this may not be assumed from the gestation at which loss occurred, whether a pregnancy was planned, or who was pregnant. Additionally, people may identify even less visible reproductive losses (e.g. assisted conception attempts that have not resulted in pregnancy) as perinatal loss and potentially as a loss of a child.

### Limitations

All eligible studies came from the Global North. As identified by Pyle et al. (19), sample characteristics reflect the ways in which power structures affect who is more able to access assisted conception and healthcare, and this is evident in the lack of diversity of participant characteristics within the samples in the included studies. We recognise that the views of those who are additionally discriminated against in relation to race, ethnicity, education and income have been less heard within the included studies and therefore our analysis. Similarly, the views of people who have not accessed any support are less likely to have been included, given that recruitment to all included studies was predominantly via LGBTQ+ organisations and bereavement organisations, and we recognise this as a wider challenge within research. This may have influenced our findings as the views of people either who did not feel the need to access to support, or who felt particularly unable or unsafe to access needed support are less likely to have been included. We note too that no participants were identified as being intersex in any of the included studies. Study 5 was the only study to comment on this and therefore with the other studies it is not known whether this reflects what was asked of participants, or who took part. This leaves us unable to comment on the possible implications of how endonormativity may operate alongside cis and heteronormativity. In hindsight, although our use of truncation (i.e. LGB*) will mean that expansive variants would not have been missed by our search strategy (e.g. LGBTQ2S+ or LGBTQIAPNb+), it was an omission for us not to have included a wider range of specific terms (e.g. intersex). Lastly, we identified but did not include one undergraduate thesis and one doctoral thesis that met most eligibility criteria, although we did include two papers published from the doctoral thesis. This decision was made to ensure quality of included literature, but may have meant that some research findings were not examined.

## Conclusion

LGBTQ+ people’s perinatal loss experiences share parallels with existing evidence with cisgender heterosexual women who have been pregnant and with cisgender heterosexual men whose partners have been pregnant. Various aspects cohere with wider evidence on loss in the context of assisted conception. Additionally, this review finds that the psychological impacts of perinatal loss in LGBTQ+ people may be amplified by cisheteronormative systems and interactions, both within services and individuals’ own networks, and that related feelings of disenfranchisement may be considerable and accompanied by isolation. Further research is needed to evaluate individual-based support for people who have held gestational roles, non-gestational roles or both, and couples-based support. Support and its evaluation should include consideration both of subsequent reproductive choices and of experiences in any subsequent pregnancies, birth and early parenting.

## Data Availability

The original contributions presented in the study are included in the article/**Supplementary Material**. Further inquiries can be directed to the corresponding author/s.
