# Supplementary material for: Enduring hope and loss: qualitative evidence synthesis of LGBTQ+ experiences of perinatal loss
Source: Front Psychiatry. 2026 Jan 16;16:1732197. doi: 10.3389/fpsyt.2025.1732197 (PMC12856493; doi:10.3389/fpsyt.2025.1732197)
Supplement: Supplementary file 1 [file Supplementaryfile1.docx]

**Supplementary File 1 Records that were excluded at full text, with reasons**

| **Reference** | **Reason and any other key information** |
| --- | --- |
| Hampson, E. 2022. Partners and Pregnancy Loss: Perspectives from Co-mothers in the UK. Prof Doc Thesis, University of East London School of Psychology <https://doi.org/10.15123/uel.8v644> | Thesis  Interviews with 5 co-mothers (which is confirmed in the text as meaning non-pregnant partners in female same-sex relationships) |
| Rose, A. 2020. Adding More Layers to Loss: LGBTQ+ People’s Experiences of Pregnancy Loss. Bachelor of Psychological Science (Honours) Thesis, The University of Adelaide. Available at: <https://digital.library.adelaide.edu.au/dspace/bitstream/2440/131285/1/RoseA_2020_Hons.pdf> | Thesis; the published paper has been included |
| Wojnar, D.M. 2005. Miscarriage experiences of lesbian birth and social mothers. PhD Thesis, University of Washington. Available at: <https://www.proquest.com/docview/305422136/?sourcetype=Dissertations%20&%20Theses> | Thesis; the published paper has been included |
| Craven, C. & Peel, E. (2014) Stories of grief and hope: Queer experiences of reproductive loss. In M.F. Gibson (Ed.) Queering Maternity and Motherhood: Narrative and theoretical perspectives on queer conception, birth and parenting. (pp. 97-110) Bradford, Ontario: Demeter Press. <https://repository.lboro.ac.uk/articles/chapter/Stories_of_grief_and_hope_Queer_experiences_of_reproductive_loss_/9478817?file=17103605> | Book chapter, draws on two studies but not reporting as empirical study |
| Peel, E., & Cain, R. (2012) 'Silent' Miscarriage and Deafening Heteronormativity: A British Experiential and Critical Feminist Account. In: C. Komaromy & S. Earle (Eds.) Understanding reproductive loss: perspectives on life, death and fertility. (pp. 79-92) Ashgate, Aldershot: Routledge. <https://kar.kent.ac.uk/43678/> | Book chapter, not reporting as empirical study |
| Allen, K.R., Craven, C.C. (2020). Losing a Child: Death and Hidden Losses in LGBTQ-Parent Families. In: Goldberg, A.E., Allen, K.R. (eds) LGBTQ-Parent Families. Springer, Cham. <https://doi.org/10.1007/978-3-030-35610-1_22> [Losing a Child: Death and Hidden Losses in LGBTQ-Parent Families \| SpringerLink](https://link.springer.com/chapter/10.1007/978-3-030-35610-1_22) | Book chapter, not reporting empirical study |
| Sycz, L.J., Evans, A.T., Denney-Koelsch, E.M. (2023). Unique Experiences of Family Building and Perinatal Loss Using Assisted Reproductive Technologies with Heterosexual and LGBTQ+ People. In: Denney-Koelsch, E.M. (eds) Perinatal Bereavement Rituals and Practices Among U. S. Cultural Groups. Springer, Cham. <https://doi.org/10.1007/978-3-031-47203-9_10> [Unique Experiences of Family Building and Perinatal Loss Using Assisted Reproductive Technologies with Heterosexual and LGBTQ+ People \| SpringerLink](https://link.springer.com/chapter/10.1007/978-3-031-47203-9_10) | Book chapter, not reporting empirical study |
| Black, Beth Perry PhD, RN; Fields, Wendy Smith MSN, APRN, FNP-C. Contexts of Reproductive Loss in Lesbian Couples. MCN, The American Journal of Maternal/Child Nursing 39(3):p 157-162, May/June 2014. \| DOI: 10.1097/NMC.0000000000000032 [MCN: The American Journal of Maternal/Child Nursing](https://journals.lww.com/mcnjournal/Abstract/2014/05000/Contexts_of_Reproductive_Loss_in_Lesbian_Couples.6.aspx) | Literature review/position piece for practitioners – not reporting empirical study |
| Janiak, E., Braaten, K. P., Cottrill, A. A., Fulcher, I. R., Goldberg, A. B., & Agénor, M. (2021). Gender diversity among aspiration-abortion patient. *Contraception, 103*(6), 426-427. <https://doi.org/10.1016/j.contraception.2021.01.013> | Does not report experiences; quantitative survey to describe gender identities of people accessing termination of pregnancy services. |
| Everett, B. G., Kominiarek, M. A., Mollborn, S., Adkins, D. E., & Hughes, T. L. (2019). Sexual orientation disparities in pregnancy and infant outcomes. Maternal and child health journal, 23, 72-81. <https://doi.org/10.1007/s10995-018-2595-x> | Does not report experiences; quantitative analysis of national survey to investigate inequities in pregnancy and birth outcomes by sexual orientation |
| Charlton, B. M., Everett, B. G., Light, A., Jones, R. K., Janiak, E., Gaskins, A. J., ... & Austin, S. B. (2020). Sexual orientation differences in pregnancy and abortion across the lifecourse. Women's Health Issues, 30(2), 65-72. <https://doi.org/10.1016/j.ajog.2013.06.036> | Does not report experiences; quantitative analysis of cohort study datasets to investigate inequities in pregnancy outcomes by sexual orientation |
| Ferrara, I., Blet, R., & Grudzinskas. (2000) Intrauterine donor insemination in single women and lesbian couples: a comparative study of pregnancy rates. Human Reproduction, 15(3):621-5 <https://doi.org/10.1093/humrep/15.3.621> | Does not report experiences of loss; quantitative analysis of fertility treatment outcomes which includes comparative miscarriage data for single women and partnered lesbians |
