# Supplementary material for: Enduring hope and loss: qualitative evidence synthesis of LGBTQ+ experiences of perinatal loss
Source: Front Psychiatry. 2026 Jan 16;16:1732197. doi: 10.3389/fpsyt.2025.1732197 (PMC12856493; doi:10.3389/fpsyt.2025.1732197)
Supplement: Supplementary file 2 [file Supplementaryfile2.docx]

**Supplementary File 2 Characteristics of included studies (n=7, reported in 10 papers)**

| **Study ID, first author, year, country,**  **Aim** | **Sample characteristics (reflecting language used in papers)** | **Data collection and analysis** | ***Paper (if multiple papers from study)*** | **Relevant findings and/or recommendations** |
| --- | --- | --- | --- | --- |
| 1. Andalibi, 2021, USA – reported across 3 papers | *Sample overview:*  17 LGBTQ+ people  *Relationship to loss:*  14 “physically experienced pregnancy loss”, 3 “in an intimate partnership in which pregnancy loss occurred”  *Gender:*  15 cisgender women, 1 transmasculine person, 1 non-binary person  *Sexual orientation:*  3 lesbian, 1 bisexual, 5 Queer, 2 asexual, 6 multiple orientations  *Race/Ethnicity:*  Race: 13 White, 1 Black/African American, 1 Latinx, 1 multiple races/ethnicities, 1 human  Ethnicity: 1 African American, 7 White, 2 American, 1 Jewish, 2 Latinx, 3 European, 1 multiple  *Country:*  All USA  *Age:*  Mean 34.4 yrs (SD 3.3)  *Relationship details:*  16 married, 1 single  *Loss details*  -single losses reported  - pregnancy loss (defined as an undesired loss of a pregnancy at any gestational age) in last 2 years  - gestation at loss n/r  *Conception details:*  n/r  *Parity:*  n/r  *Other notes:*  Information on education, income, religion, geography, social media use available in papers  *Recruitment:*  Personal networks and social media networks | Semi-structured interviews  Conducted remotely according to participant preference of phone or videocall, with option for camera to be on or off  Duration 27-97 mins (mean 67 mins, SD 21)  Thematic analysis  The 3 different papers offer different approaches to the findings: organising through the Disclosure Decision Making (DDM) Framework (1a), multi-level stigma and resilience theories (1b), intracategorical intersectionality as a foundational framework and focused on benefits and challenges of being in particular online spaces (1c) | 1a. Pyle, 2021, USA.(19)  Aim: to investigate (non)disclosure of pregnancy loss among LGBTQ people to known ties on social media | Findings  - the Disclosure Decision Making Framework (DDM) can explain LGBTQ people’s decisions to disclose pregnancy loss to social media connections, highlighting the role of intersectional stigma  - self-related factors included individual personality and self-help mechanisms  - audience-related factors included concerns that revealing the loss would lead to intrusive questions about LGBTQ identity, questions related to transition, or method of conception, and also fears of homophobia and/or transphobia, making disclosure less likely  - network-related factors included the desire to specifically support other LGBTQ people dealing with loss, in recognition that many LGBTQ people had undisclosed losses, making disclosure more likely  - societal factors included wider societal stigma with some participants choosing to disclose to increase visibility of LGBTQ pregnancy loss  - platform and affordance factors included lack of control over information on social media and a fear of sensationalism  Recommendations  - social media platforms can facilitate disclosure about ‘silenced topics’ in various ways (e.g. enabling selective disclosure, providing education, prioritising disclosures in news feeds) |
|  |  |  | 1b. Lacombe-Duncan, 2022.(20)  Aim: informed by minority stress theory, to explore the experiences of multi-level stigma and resilience among LGBTQ+  people in the context of conception, pregnancy, and loss | Findings  - people linked the unique challenges of conceiving for LGBTQ+ people (including medical, financial and emotional processes) with fear of pregnancy loss and with devastation when pregnancy loss occurred  - stigma is multi-level: intrapersonal (anticipated and internalised sexual stigma about LGBTQ+ people being parents; internalised infertility and/or pregnancy loss stigma), interpersonal (being asked inappropriate/invasive questions about conceiving as LGBTQ+ people; unsolicited advice/judgement about conception and pregnancy process; cisnormative and/or heteronormative assumptions; intersecting interpersonal stigma e.g. with race or relationship status), and structural (cisnormativity and heteronormativity embedded in systems; lack of LGBTQ+-specific services and resources, particularly intersectionally-affirming; cost/lack of coverage)  - resilience is multi-level: individual (attending to one’s emotions in response to pregnancy loss; asking for what one needs, knowing limits, and setting boundaries; purposeful disclosure), relational (LGBTQ+-specific fertility/pregnancy online groups; connecting specifically around pregnancy loss with other LGBTQ+ community members; partner support; in-person family/friend support; healthcare and other provider support), and collective (development and availability of LGBTQ+-specific and intersectionally-affirming support spaces; importance of LGBTQ+-specific groups for feeling seen and protected and having trust; broad commitment to share information, affirmation, and narration of underrepresented stories)  - aspects of resilience may help to mitigate against negative impacts of minority stressors (i.e. stigmas)  Recommendations  - minority stress theory can be used to understand “strengths-based and affirming approaches to reproductive healthcare for LGBTQ+ people”  - structural change is needed including training for healthcare providers, materials that are specific to LGBTQ+ perinatal loss and are intersectionally-affirming (e.g. different abilities, ethnicities), support online and offline, and policy change to address differential access to conception |
|  |  |  | 1c. Andalibi, 2022.(21)  Aim: informed by an intracategorical intersectional lens, to uncover the benefits and challenges of LGBTQ-specific and non-LGBTQ-specific pregnancy and loss-related online spaces | Findings  - Theme 1a. benefits of LGBTQ-specific online spaces were enacting individual, interpersonal, and collective resilience through shared sense of identity and experience leading to a supportive, inclusive, affirming and safe online community, where people could ask and answer questions and could connect with smaller branches of the community that shared multiple aspects of identities and experiences  - Theme 1b. challenges of LGBTQ-specific online spaces were lack of representation and visibility of identities and/or experiences where assumptions were still made, whereby people with multiple marginalised identities (e.g. people of color and non-partnered individuals) faced greater barriers to finding support in LGBTQ-specific spaces compared to those with privileged identities (e.g. White and married).  - Theme 2a. benefits of non-LGBTQ-specific online spaces concerned the ability to meet some more general informational needs  - Theme 2b. challenges of non-LGBTQ-specific online spaces concerned heteronormativity, cisnormativity, and lack of understanding, coupled with a perceived need for LGBTQ+ people to educate, which collectively prevented community and emotional needs from being met  Recommendations  - more equitable and inclusive environments are needed for LGBTQ people both in reproductive healthcare context and in non-LGBTQ-specific online spaces relating to pregnancy and loss  - social media design should consider intersectional aspects, e.g. facilitating nested identity-based spaces, with consideration of who provides moderation |
| 2. Cacciatore 2011, USA.(22)  Aim: to explore child  death in same-gendered-parent families; specifically ‘maternally bereaved lesbians’. | *Sample overview:*  6 lesbian mothers  *Relationship to loss:*  Not specified – describes as maternally bereaved lesbians  *Gender:*  Language of women is used throughout, without any mention of cisgender/transgender  *Sexual orientation:*  All lesbian  *Race/Ethnicity:*  Ethnicity: 4 Caucasian, 1 Jewish, 1 Italian-Irish, 1 European-Canadian  *Country:*  *USA*  *Age:*  3 aged 36-45, 3 aged 46-55  *Relationship details:*  4 partnered (including 1 married in Canada), 2 single  *Loss details*  - 5 “of the children had died during the perinatal period or birth”, 1 had died in adulthood; describes “all the deaths were traumatic and unexpected”  -details of any earlier losses n/r  -time since loss n/r  *Conception details:*  - n/r  *Parity:*  - inclusive of losses, 2 had 1 child, 3 had 2 children, 2 had 3 children; 2 had no living children at the time of participation  Other notes:  Information on religion and socioeconomic demographics available in paper  *Recruitment:*  Snowball sampling beginning with bereavement organisations, gay and lesbian parenting and pride organisations, gay and lesbian media including newsletters and magazines | Multiple-case study design, using semi-structured interviews (2-4hr, plus 1 being 5 hrs)  Constant comparative analysis | Findings  - findings were presented in the following sections (although not described as e.g. ‘themes’): ‘the death and bereavement experience’, ‘social support and disenfranchisement’, ‘rebuilding, reintegration, and reflection’  - authors reported that, “overall, the experiences of bereaved lesbian parents were more similar to those of heterosexual bereaved mothers than different”  - not fitting in with lesbians (because a mother), with bereaved parents (because a lesbian), with mothers (because a lesbian and bereaved)  - experiences of poor care were experienced by most, linked to staff discomfort; one experience compassion from medical staff and found this meaningful and helpful  - partners not recognised on birth or death certificates  - perinatal loss is a physical as well as emotional experience  - recognising that getting pregnant is not as easy for lesbians as for most heterosexual couples  - loss has permanently changed their identity  - links to literature on ‘disenfranchised grief’ and frames experiences of lesbian maternal bereavement as a type of ‘double-disenfranchisement’ and that social support may be insufficient to meet psychological needs  Recommendations  - Various listed for practice, including explicitly including LGBT+ families in policies and language, free of heterosexual language; providing appropriate resources that recognise different families; consider mental health; consult with LGBT+ communities  - Further research | |
| 3. Peel, 2010, UK, USA, Canada and Australia.(23)  Aim: to explore lesbian and  bisexual women’s experiences of miscarriage, stillbirth and neonatal death. | *Sample overview:*  60 women  *Relationship to loss:*  78% had “physically experienced their loss(es) (i.e. carried the pregnancy)” (also referred to as “birth mothers”), 22% “had experienced loss as the social mother (i.e. the partner of the women who had carried the pregnancy)”  *Gender:*  Language of women is used throughout, without any mention of cisgender/transgender  *Sexual orientation:*  All described as non-heterosexual; 76.6% were lesbian, 15% bisexual, 8.3% ‘other’  *Race/Ethnicity:*  92% white (further details n/r)  *Country:*  43.3% UK, 28.3% USA, 18.3% Canada, 10.0% Australia  *Age:*  Mean 35, range 22-55 years  *Relationship details:*  82% in relationship with a woman, 8% single, 5% in polyamorous relationships, 3% married to a man, 1 in a relationship with a trans man. (Note: this is current relationship; 90% were in same relationship at time of loss)  *Loss details:*  - one-third had experienced multiple losses  - majority early miscarriage, some late miscarriage, stillbirth or neonatal death  - majority experienced most recent loss occurred in past 5 years (83%), including 45% of sample in past year  *Conception details:*  - all except one were planned pregnancies  - 84% conceived using donor sperm (majority at a clinic, some at home, minority via IVF); 14% via sexual intercourse with male partner, 2% via sexual intercourse with a man who was not their partner  - conceived within 1 month-10 years of trying (mean 9.2 months, SD n/r)  *Parity:*  -unclear: reports that 55% had children, with mean age 4.5 years (range 4 days-17 years); also reports number of losses  Other notes:  Information on social class and disability available in paper  *Recruitment:*  Strategic opportunistic sampling; recruitment emails distributed via various distribution lists (LGBT and academic) and publicised via community organisation website in UK, USA and Canada; invitations were also sent to “mainstream” miscarriage, stillbirth and neonatal death organisations but they declined to publicise the study. | Online qualitative survey  Online survey  Descriptive statistics for closed questions and thematic analysis for open-ended qualitative responses, organising into the “most prevalent themes” | Findings  - Three themes: ‘processes and practices for conception’, ‘amplification of loss’, ‘health care and heterosexism’  - 71% rated standard of care from health professionals ‘good’, ‘very good’ or ‘outstanding’, 17% rated their care as ‘poor’, ‘very poor’ or ‘extremely poor’  - 26.8% said they had experienced homophobia, heterosexism or prejudice from a healthcare provider and 8.6% were ‘unsure’  - ‘experience of loss was amplified due to contextual factors and the investment respondents reported making in impending motherhood’  Recommendations  - health professionals should not assume heterosexuality and should ensure that same-sex partners are “acknowledged and actively included”  - procedural changes are needed e.g. changing forms (e.g. gender of partner)  - collect sexual orientation of patients attending clinics and maternity services  - make non-heterosexual women more visible in reproductive health and pregnancy loss research, including couples and single people | |
| 4. Riggs, 2020, Australia, USA and European Union.(24)  Aim: to explore experiences of  pregnancy loss among a sample of men, trans/masculine,  and non-binary people who had undertaken a  pregnancy | *Sample overview:*  16 men, trans/masculine, and non-binary people  *Relationship to loss:*  All had experienced pregnancy loss, “having undertaken at least one pregnancy following a gender transition”  *Gender:*  Collectively described as men, trans/masculine and non-binary; multiple genders listed; most used he/him or they/them pronouns  *Sexual orientation:*  Range of sexual orientations (e.g. bisexual, fluid, gay, human-sexual, pansexual, queer), including multiple stated; 2 n/r; 0 stated heterosexual  *Race/Ethnicity:*  n/r  *Country:*  3 Australia, 8 European Union (including UK), 5 US  *Age:*  Mean 35, range 23-49  *Relationship details:*  2 single/no partner, 1 casual partners, 13 in a relationship (including 1 now separated; 4 with cisgender woman, 6 with cisgender man, 3 with transgender man)  *Loss details*  -10 had one pregnancy loss, 6 had multiple  - Gestational age at loss ranged from 3-23 weeks; authors described as 19 early term losses (12 weeks or less) and 5 late term losses.  -Time since loss n/r  *Conception details:*  - n/r  *Parity:*  Separate to the pregnancy losses, 13 had live births (range 1-4) and the remaining 3 were pregnant at time of interview  Other notes:  Taken from a wider trans masculine pregnancy project involving 51 participants; 16 of whom had experienced loss  *Recruitment:*  Personal networks, social media networks and community members | Semi-structured interviews, either in person or via videocall (1-3 hours, mean 100 mins)  Thematic analysis | Findings  - 10 themes: (1) pregnancy losses count as children, (2) minimizing pregnancy loss, (3) accounting for causes of pregnancy loss, (4) pregnancy loss as devastating, (5) pregnancy loss as having positive meaning, (6) fears arising from a pregnancy loss, (7) experiences of hospitals enacting inclusion, (8) lack of formal support offered, (9) lack of understanding from family, and (10) importance of friends  - some similarities with research with cisgender men and cisgender women following pregnancy loss, including strong emotional responses (common with both) but unlike research with cisgender men, did not report experiencing expectation of needing to be stoic, which may be linked to them having undertaken gestational role  - some participants experienced pregnancy loss as “distressing, but also as a sign that their bodies were working”, which the authors frame as contrary to a ‘failed body’ narrative  - as well as some similarities, also ‘unique points of difference’ including need for inclusive healthcare (e.g. asking about pronouns, refusing to accept misgendering in systems), specific meanings that may be brought to the pregnancy loss (e.g. concerns about testosterone), how marginalisation may affect available support  Recommendations  - clinicians need to focus on emotions attached to the loss and possible desire to attempt another pregnancy, not on pregnancy loss as inferring their body should not be pregnant  - clinicians need to acknowledge that for some people, pregnancy loss may be experienced as loss of a child, warranting supportive counselling for both “gestational parents and their partners”  - may involve family members in counselling  - need for systems to correctly record pronouns and gender – to avoid compounding potential grief (including experienced by partners) | |
| 5. Rose, 2022, 2023, Australia and England – reported across 2 papers | *Sample overview:*  14 LGBTQ+ people  *Relationship to loss:*  - 5 experienced pregnancy loss as gestational parents (only)  - 4 experienced pregnancy loss as both gestational parents and as non-gestational parents (where a partner was pregnant)  -5 cisgender men experienced pregnancy loss as non-gestational parents in context of surrogacy  *Gender:*  8 cisgender women, 5 cisgender men, and 1 trans non-binary person; although eligible, 0 intersex or asexual people took part  *Sexual orientation:*  Reported as a range represented within LGBTQ+; 11 were in a same-gender relationship at the time of loss  *Race/Ethnicity/‘Cultural diversity’:*  -includes 2 Aboriginal and Torres Strait Islander (self-describing as Caucasian/Indigenous and Aboriginal-mixed), 1 Greek-Australian, 1 Irish, 1 4^th^ generation Australian, 1 English, 1 Anglo-Australian, 2 Anglo-Celtic, 3 White, 2 n/r (detail taken from thesis)  -2 had partners for whom English was a 2^nd^ language  *Country:*  Australia (12) and England (2)  *Age:*  30-60 years (mean 40)  *Relationship details:*  All in long-term relationships  *Loss details*  - 2 experienced 1 pregnancy loss; 12 experienced multiple (2-5)  - details on gestational age at loss n/r; includes “ectopic pregnancies, miscarriage, medically induced termination and stillbirth”  -notes that some also experienced “other reproductive losses, including unsuccessful egg insemination or embryo transfers”  - time since loss: 8m-10years (mean 3.78yrs, SD 2.59)  *Conception details:*  - n/r  *Parity:*  - 6 caring for living children at the time of interview (details n/r; thesis reports 8 have living children)  Other notes:  Paper 5b is limited to a subset of the 12 participants residing in Australia. Information on surrogacy details available in papers. Further sample details available in thesis (including religion).  *Recruitment:*  Email and social media networks of multiple organisations relating to LGBTQ+ conception, surrogacy and parenting, LGBTQ+ mental health, pregnancy loss support, and perinatal mental health | Semi-structured interviews, in person or via telephone or videocall (47-127min, mean 87, SD 11.09)  Thematic analysis | 5a. Rose & Oxlad, 2022.(25)  Aim: to explore how the context in the lead up to loss was  important for LGBTQ+ people when pregnancy loss occurred | Findings  - One superordinate theme of ‘The context of societal attitudes to  LGBTQ+ people and their efforts to conceive are essential to understanding LGBTQ+ peoples’ grief and support experiences after pregnancy loss’, within which were 3 themes.  - 1. ‘The complexity of non-normative decision-making’ including aspects considered unique to LGBTQ+ people, questions of personal identity, beliefs and norms about kinship, morality and LGBTQ+ deservedness, complex decision-making (where applicable) on how to create families including who wants to and can carry the pregnancy, potentially reconsidering options when loss(es) occurred, and ‘trade-offs’ between options  - 2. ‘Outgroup distress and marginalization’ including repeated exposure to minority stressors prior to loss, anticipated and experienced discrimination and exclusion during reproductive healthcare, repeated ‘coming out’ which may not feel safe, heteronormative attitudes impacting pregnancy loss experiences, needing to (re)engage with services that created distress  - 3. ‘Resource depletion’ describing high levels of physical, emotional,  social, time and financial investment in conception that depleted resources prior to loss, impacting on ability to manage or cope with loss experiences  Recommendations  - Need for inclusivity training for healthcare professional and awareness of context of conception and potential impact on pregnancy loss experiences  - Need for visible indicators of inclusivity and inclusive language in healthcare settings |
|  |  |  | 5b. Rose & Oxlad, 2023.(26)  Aim: to explore LGBTQ+ people’s workplace leave and support experiences following pregnancy losses as gestational or non-gestational parents | Findings  - Participants’ experiences of work were described as key aspects of their overall pregnancy loss experience and therefore relevant to this review  - Theme 1 - Disclosure in the workplace: support, shame, and self-protection (with subthemes of: seeking support in the workplace, engaging in self-protection in the workplace)  - Theme 2 - Navigating discriminatory workplace policies, leave, and entitlements  - Theme 3 - Coping at work: getting the balance right for each person (variation in return to work, with most describing returning to work within 1-7 days of pregnancy loss as a way to manage their grief, and shaped by internalised norms minimising and stigmatising grief following pregnancy loss).  - Heteronormative policies and entitlements can compound LGBTQ+ people’s distress relating to pregnancy loss, including leave entitlements (access, misgendering on forms), not accommodating or recognising the bonding that may happen by an early point of gestation and the distance that may be involved when using surrogacy.  Recommendations  - Employers need to use inclusive language in leave policies and forms, provide parental and bereavement leave to people of all genders and sexualities, regardless of family formation method and gestational age  - There should be the option for flexibility in hours, workload, and tasks following pregnancy loss and there should be flexibility where people initially return to work but then desire or require leave |
| 6. Røseth, 2023, Denmark and Norway.(27)  Aim: to explore how Danish and Norwegian lesbian couples bereaved of a  child in the perinatal period experience the loss, and to examine the maternity care  offered | *Sample overview:*  6 lesbian couples  *Relationship to loss:*  6 birth mothers/biological mothers and 6 non-birth mothers/co-mothers  *Gender:*  Language of women is used throughout, without any mention of cisgender/transgender  *Sexual orientation:*  All lesbian  *Race/Ethnicity:*  n/r  *Country:*  4 couples Danish nationality and living in Denmark, 2 couples Norwegian nationality and living in Norway  *Age:*  29-42 years  *Relationship details:*  “Living in a relationship with another woman when they lost their baby”  *Loss details*  - gestation at loss 21-40 weeks (eligibility was “late pregnancy or the first few days after birth” and some included birthing a baby who “had already died or risked dying”)  - losses occurred within last 3 years  *Conception details:*  - n/r  *Parity:*  - n/r  Other notes:  None  *Recruitment:*  Social media networks for (Facebook) bereavement groups and LGBTQ groups, and via midwives working with bereavement groups | Semi-structured interviews with couples, in person or via videocall (63-94min)  Descriptive phenomenology | Findings  - Overall meaning structure: strong desire to become a family with emotional investment in the pregnancy, where the birth of their dead baby was experienced as extremely difficult, stressful, and vulnerable, with a need for care and support from experienced healthcare professionals to create a meaningful space to give birth (examples given about the importance of being cared for midwives, and on maternity wards, with the ability to give caring rituals to their baby, with language being about birthing a child rather than aborting a fetus);  - This was divided into 3 inter-related constituents  - 1. ‘Developing queer parenthood through creating a meaningful space to give birth’ - this included the importance of both being recognised as the baby’s parents, “particularly for co-mothers, even if this meant being a parent of a dead child” and “allowing time to bond as well as to cry”; although none felt they were discriminated, some described sub-optimal care from some healthcare professionals (e.g. co-mothers being excluded by forms or in interactions, assumptions being made, irrelevant questions being asked)  - 2. ‘Enabling love and beauty by giving time and space after the birth’ - this included spending time with the baby as helping them to bond with their baby; some felt being two women meant the co-mother found it easier to understand what her partner was going through but noted differences between the roles, with bodily experience; also spoke about importance of openness and dialogue in their relationships  - 3. ‘Staying connected while living on’ - this included complexity about whether to choose a different donor if there had been a genetic aspect to the baby’s death, or to decide for the partner to pursue the next pregnancy  - co-mothers’ relationships to their children – and therefore their losses – were less recognised by healthcare systems, legal systems and society, leaving them more vulnerable to disenfranchised grief  Recommendations  - healthcare personnel need to be informed of continued heterosexual normativity (including through forms used) and move beyond suboptimal care or insensitive comments, and to instead sensitively acknowledge the specific needs of queer couples, to enable them to be present in them moment, process their loss and develop an identity as a parent  - need for bereaved lesbian couples to be able to meet with others in similar situations | |
| 7. Wojnar, 2007, USA and Canada.(28)  Aim: explore miscarriage amongst lesbian couples | *Sample overview:*  10 lesbian couples  *Relationship to loss:*  10 ‘birth mothers’(“the partner who conceived and miscarried pregnancy”) and 10 ‘social mothers’ (“the birth mother’s female partner”)  *Gender:*  Language of women is used throughout, without any mention of cisgender/transgender  *Sexual orientation:*  All lesbian  *Race/Ethnicity:*  All white  *Country:*  USA and Canada  *Age:*  30-45 (birth mothers: mean 37.6, SD 4.19, range 30-42; social mothers: mean 38.0, SD 4.60, range 31-45)  *Relationship details:*  In ‘committed’ relationship (at time of loss and still in that relationship)  *Loss details*  - 1-4 miscarriages  - gestation at loss 5-20 weeks, mean 11.0 weeks, SD 4.34  -losses occurred 1 week-2 years prior to study enrolment, mean 11.6 weeks, SD 4.25  *Conception details:*  - 5 used ‘alternative insemination’ with known donor, 5 used unknown donor; n/r whether clinic or home  *Parity:*  - 0-3 living children: previous to loss, 5 birth mothers had given birth ,0 social mothers had given birth  Other notes:  Information on education and employment available in paper  *Recruitment:*  Publicised via newspapers, online and “posted in other areas” | Series of 3 face to face interviews per couple: one with each partner (2 hours), one with couple 2-3 weeks later (1 hour)  Descriptive phenomenology | Findings  - overarching theme – “We are not in control” (representing a loss of control both concerning conception and loss where these are intertwined experiences and “cannot fully comprehend lesbians’ experiences of miscarrying without understanding their difficulties conceiving”)  - Subtheme 1 “We work so hard to get a baby” (with three further aspects concerning the “hard work” involved in planning and becoming pregnant that compound experiences of miscarriage: Do we want to be mothers? The up and down ride. Can you see the heartbeat?)  - Subtheme 2 “It Hurts So Bad: The Sorrow of Miscarriage” (describing miscarriage as an unanticipated and painful experience; with 5 processes described: The “hope-no-hope” ride; Living through the crash; We were pregnant and now we are not; Clarifying what matters most; Moving on)  - birth mothers experienced enduring physical and emotional pain  - social mothers were commonly not recognised by others as being grieving parents  - birth mothers usually grieved their loss openly whereas most social mothers felt they needed to be strong for their partners and kept their sadness more private  - some social mothers became caregivers to their partners and facilitated expression of grief through rituals but others felt paralysed and uncertain what to do  Recommendations  - need for practitioners to be aware of and sensitive to lesbian couples’ unique perspectives  - need for practitioners to acknowledge the pregnant and non-pregnant partner, including potential for different experiences and grief responses; acknowledge social mother as a grieving parent | |
